# New combined absorption/^1^H NMR method for qualitative and quantitative analysis of PET degradation products

**DOI:** 10.1007/s11356-024-32481-0

**Published:** 2024-02-23

**Authors:** David Kornberger, Tanja Paatsch, Magnus Schmidt, Ulrike Salat

**Affiliations:** 1 Faculty Medical and Life Sciences, Institute of Applied Biology, Molecular Biology Lab, Furtwangen University, Jakob-Kienzle-Str. 17, 78054 Villingen-Schwenningen, Germany; 2 Faculty Medical and Life Sciences, Institute of Precision Medicine, Organic and Bioorganic Chemistry Labs, Furtwangen University, Jakob-Kienzle-Str. 17, 78054 Villingen-Schwenningen, Germany

**Keywords:** Biodegradation, PETase, MHETase, *Ideonella sakaiensis*, ^1^H NMR analysis, Bulk absorbance measurement, Mono(2-hydroxyethyl) terephthalic acid, Bis(2-hydroxyethyl) terephthalic acid, Terephthalic acid

## Abstract

Poly(ethylene terephthalate) (PET) is a very valuable and beneficial material for industrial purposes, with various different applications. Due to the high annual production volume of over 50 million tons worldwide and the indiscriminate disposal by consumers, the polymers accumulate in the environment, causing negative effects on various ecosystems. Biodegradation via suitable enzymes represents a promising approach to combat the plastic waste issue so validated methods are required to measure the efficiency and efficacy of these enzymes. PETase and MHETase from *Ideonella sakaiensis* are suitable enzymes needed in combination to completely degrade PET into its environmentally friendly monomers. In this project, we compare and combine a previously described bulk absorbance measurement method with a newly established ^1^H NMR analysis method of the PET degradation products mono(2-hydroxyethyl) terephthalic acid, bis(2-hydroxyethyl) terephthalic acid and terephthalic acid. Both were optimized regarding different solvents, pH values and drying processes. The accuracy of the measurements can be confirmed with sensitivity limits of 2.5–5 µM for the absorption method and 5–10 µM for the ^1^H NMR analysis. The combination of the described methods therefore allows a quantitative analysis by using bulk absorption coupled with a qualitative analysis through ^1^H NMR. The methods established in our work can potentially contribute to the development of suitable recycling strategies of PET using recombinant enzymes.

**Supplementary Information:**

The online version contains supplementary material available at 10.1007/s11356-024-32481-0.

## Introduction

The background to the objective is the plastic waste problem in the environment and the potential solution of biodegrading the polymers by plastic-degrading enzymes. In this work, the focus is on the plastic poly(ethylene terephthalate) and on the measurements of its degradation products produced by biodegradation via PETase und MHETase.

### Biodegradation of poly(ethylene terephthalate)

PET is one of the most abundant plastics worldwide with a global production volume of over 50 million tons per year (Dissanayake and Jayakody 2021). PET is mainly used to produce beverage bottles, films and polyester textiles. Its durability and high resistance to natural degradation are a major drawback for recycling processes, making them very costly and energy intensive. Plastic waste accumulates in the environment due to poor waste treatment and poses a major threat to various ecosystems (Law et al. 2020). As plastics accumulate, microorganisms adapt and develop enzymes to break down the manmade plastics and use some of them as sources of carbon and energy (Austin et al. 2018). Biodegradation by optimized enzymes may be a solution to combat the plastic waste problem. PET, for example, has a glass transition temperature above 70 °C, at which the polymer becomes more amenable to enzymatic attack, thus ensuring more efficient degradation at a correspondingly high reaction temperature. Consequently, the optimization of enzymes through protein engineering of the amino acid residues involved in the thermostabilization of polyester hydrolases represents a potential approach for industrial PET recycling processes. (Wei and Zimmermann 2017a).

In principle, many microorganisms are associated with the biodegradation of plastics, but most enzymes involved have not yet been identified. Therefore, the aim of research is to identify, isolate, synthesize and optimize plastic-degrading enzymes. PET hydrolases represent the largest fraction of the enzymes studied. Involved are serine hydrolases with a typical α/β-hydrolase fold and a catalytic triad of the amino acids serine, histidine and aspartic acid (Wei et al. 2014).

### PETase and MHETase from Ideonella sakaiensis

To date, the main known plastic-degrading enzymes are responsible for the biodegradation of polyurethan, such as PueB lipase and PueA lipase from *Pseudomonas chlororaphis*, and for the degradation of poly(ethylene terephthalate), for example, by CalB lipase from *Candida antarctica* (Danso et al. 2019). In addition to enzymes from *Candida antarctica*, other hydrolases are known for PET degradation, such as enzymes from the genera *Thermobifida*, *Thermomonospora*, *Ideonella sakaiensis*, *Bacillus*, and *Saccharomonospora* (Wei and Zimmermann 2017b). The turnover rates of all known hydrolases are relatively low. For example, an enzyme from the bacterium *Thermobifida fusca* has an erosion rate of approximately 8–17 µm PET per week when incubated at 55 °C (Müller et al. 2005). Validated methods are required to measure the efficacy and efficiency of the enzymes for future optimization strategies.

In 2016, Japanese researchers discovered the bacterium *Ideonella sakaiensis* in the sediment of a PET bottle recycling plant. *Ideonella* can use PET as its main carbon and energy source of growth. *I. sakaiensis* produces two enzymes that are required in combination to completely degrade PET into its environmentally compatible monomers. The first enzyme PETase (EC 3.1.1.101) degrades PET into mono(2-hydroxyethyl) terephthalic acid (MHET) with minor amounts of terephthalic acid (TPA) and bis(2-hydroxyethyl) terephthalic acid (BHET). MHET is subsequently hydrolysed by the second enzyme MHETase (EC 3.1.1.102) into the monomers TPA and ethylene glycol (EG). Depending on the substrate, the optimal conditions are pH 7–9 and a temperature between 30 °C and 40 °C (Yoshida et al. 2016).

### Genetic approach to optimize PETase from Ideonella sakaiensis

One of the most effective and promising enzymes for the biodegradation of PET is the enzyme PETase. PETase from *I. sakaiensis* showed up to 120 times higher activity against PET films than previously tested PET-degrading enzymes. Depending on the substrate, the optimal conditions are pH 7–9 and a temperature between 30 °C and 40 °C (Yoshida et al. 2016). Ever since the discovery, research is focused on improving its thermostability, activity and efficiency. For example, the research group led by Ma et al. focused on the development and investigation of new highly efficient PETase mutants created by protein engineering of key hydrophobic sites, resulting in 2.5-fold higher PET degradation activity at 30 °C compared with wild-type PETase (Ma et al. 2018). The team of Son et al. was able to develop, also by protein engineering, a PETase variant with increased thermostability, which showed a 14-fold higher degradation activity at 40 °C compared to the wild-type PETase (Son et al. 2019). In addition, the secretion of PETase has already been improved by further development of the pelB signal peptide by random mutagenesis and screening, resulting in a 1.7-fold increase in PETase secretion, leading to more efficient hydrolysis of PET (Shi et al. 2021). The immobilization of the enzymes involved represents a further optimization opportunity regarding biodegradation.

### Analytics for biodegradation

Validated methods are required to study enzymes in terms of their efficiency and efficacy. There are already some standard methods that can be used to evaluate degradation. Visual observation using a scanning electron microscope (Yoshida et al. 2016) or similar technique provides a simple method to observe surface roughness, colour changes and the formation of cracks or biofilms on the surface (Moog et al. 2019). The loss of substrate mass can also be used for initial degradation analysis. Other analytical methods include changes in mechanical properties and molecular masses (Torena et al. 2021), CO_2_ formation and O_2_ consumption (Torena et al. 2021; Yoshida et al. 2016), enzymatic degradation (Zhong-Johnson et al. 2021), fluorescence detection methods (Wei et al. 2012) and the so-called clear zone test, which is typically used to screen for organisms capable of degrading a particular polymer (Torena et al. 2021). This study has a focus on enzymatic degradation.

The degradation products of plastic need to be analysed to determine the activity of the enzymes involved and to be able to develop suitable recycling and bioremediation strategies. We focused on the plastic PET and its degradation products MHET, BHET and TPA for the measurements. Currently, high-performance liquid chromatography (HPLC) is the most established and used method for the determination of PET degradation products, which was used, for example, by scientists such as Yoshida et al. (2016), Joo et al. (2018) and Oda et al. (2018) in their experiments. HPLC is a rapid, highly accurate and automated method for analysing samples for their chemical components, and it is highly reproducible. However, the method can be very expensive due to the large quantities of organics required and the different columns and modules. In addition, coelution can occur if two components are similar in structure and polarity, which is the case in PET degradation products, and thus elute simultaneously or almost simultaneously (Wang et al. 2021).

We evaluate a newly established ^1^H NMR analysis method as a suitable analysis method for qualification. We selected ^1^H NMR analysis, because this method is quite new regarding PET degradation products and can be performed with little effort. Furthermore, ^1^H NMR is a powerful and essential analytical tool to elucidate the structure of unknown natural and synthetic compounds resulting from the degradation of PET with respect to hydrogen-1 nuclei within the molecules (Wang et al. 2021). Since PETase degrades PET into TPA, MHET and BHET, it is necessary to detect the amount of the intermediate MHET to make a specification for MHETase, which subsequently catalyses the degradation of MHET into the monomers TPA and EG. To be able to make a statement about the quantity in addition to the qualification, we used an already described simple and rapid method that is also resource-efficient and can be performed with a low sample volume (Zhong-Johnson et al. 2021). Because this method is a bulk absorbance measurement with contributions of all degradation products to the absorbance due to the presence of the aromatic ring of TPA, MHET and BHET, it is not possible to distinguish between these products. This combined absorbance/^1^H NMR method has the great advantage that a qualitative and quantitative measurement can be carried out simultaneously with minimal sample preparation. Another advantage is the ease of performance and the low sample volume and time required, mainly with regard to the bulk absorbance method.

### Immobilization of PETase

The use of free enzymes, such as PETase, has been a challenge so far due to the complex purification processes, short life span and non-reusability. Immobilization of the enzymes can overcome these limitations. In the work of Zhu et al., a possible application of immobilized PETase for PET degradation in wastewater is described with subsequent endpoint HPLC measurement of the degradation products (Zhu et al. 2022). The innovative biofilm integrated nanofiber display (BIND) platform enables protein immobilization onto the curli of *Escherichia coli*, where the target enzyme binds with the CsgA subunit, the building block monomer of curli (Zhu et al. 2022). Consequently, in protein purification, preparation of the carrier material is not required, and the possible limitation of enzyme functionality due to physical or chemical binding is circumvented. A renewable PET-degrading biocatalyst has already been developed for plastic degradation, in which the PETase was functionally immobilized on the *E. coli* curli nanofibers, called BIND-PETase. In addition to different reaction conditions and testing regarding the storage stability and reusability of the biocatalyst, the application scenario investigated included the degradation of PET in a complex wastewater matrix with subsequent HPLC analysis. A concentration of over 4300 µM of the degradation products was measured after 15 days of reaction. Accordingly, BIND-PETase could contribute to the development of a biocatalytic strategy for advanced wastewater treatment and thus to the reduction of microplastic pollution (Zhu et al. 2022). By analysis with the combined absorbance/^1^H NMR method using flow cells for bulk absorbance and sampling at different times for ^1^H NMR, continuous monitoring of such a wastewater treatment could be achieved, allowing further conclusions on the kinetic stability of the PETase and on the product distribution in the wastewater.

## Material and methods

Chemicals and reagents were purchased from Tokyo Chemical Industry, Carl Roth, Deutero GmbH, and Sigma Aldrich and were used without further purification. Two different methods were performed to allow a combined qualitative and quantitative measurement of the degradation products TPA, MHET and BHET. In both methods, degradation products were dissolved in buffers with different pH values as well as in pure DMSO and measured in combination with heat drying, freeze drying and without drying.

### General procedure for NMR measurements

This method only involves the qualification of TPA, MHET and BHET. The individual degradation products were identified by ^1^H NMR measurements on a Spinsolve 60 Carbon Benchtop NMR spectrometer (Magritek). Chemical shift values are reported in ppm and are used as a reference depending on solvent, temperature and magnetic field. The spectra were recorded at room temperature with the 1D Proton + protocol using 8192 scans with a 90° excitation pulse (7 μs) covering a spectral range from 45 to − 36 ppm. A total of 32,000 data points is acquired with an acquisition time of 6.4 s and a repetition time of 7 s. The measurements took about 16 h. Data processing has been performed with MestReNova version 14.2.2-28739, including zeroth and first-order phase correction and baseline correction with a Bernstein polynomial fit of third order.

### General procedure for bulk absorbance measurements

To quantify the total products without differentiating between the individual degradation products, bulk absorbance was measured in 1-cm-gap UV-transparent glass cuvettes using a spectrophotometer with a 1 mL sample volume. Due to the presence of the aromatic ring, absorbance measurements were typically performed between 240 and 260 nm. For our study, the detection wavelength of 260 nm was selected, because during sample preparation, the degradation products are dissolved in dimethyl sulfoxide (DMSO), which absorbs strongly between 220 and 250 nm. Before quantifying the total products, a zero adjustment was carried out with the solvent DMSO. For each degradation product, a linear absorption profile was established with a concentration range of 100–1 µM. Duplicate determinations were made for each concentration.

### Preparation of samples

For the establishment of the linear absorption profiles, the individual degradation products were dissolved in a buffer with two different pH values (pH 9 and pH 3.54) as well as in pure DMSO to determine the optimal conditions. The value of 3.54 corresponds to the pKs of TPA. We used a 50 mM glycine–NaOH buffer containing 50 mM NaCl and 10% (v/v) DMSO. DMSO was added to solubilize the products. A final product concentration of 500 µM was obtained in each case. To further optimize the parameters, heat drying was compared with freeze drying. After drying, the degradation products were taken up into DMSO. A dilution series was prepared with the concentrations of 100, 75, 50, 25, 10, 7.5, 5, 2.5, and 1 µM. The samples were then analysed qualitatively by ^1^H NMR and quantitatively by bulk absorbance at 260 nm. In addition, a mixture of MHET, BHET and TPA with 500 µM each in buffer without drying was examined to verify the differentiation of the products by ^1^H NMR analysis.

## Results

The aim of the work was to establish a new ^1^H NMR analysis method and optimize it, as well as to optimize the absorption method already described for the measurement of PET degradation products. The methods were compared and linked to establish a new combined absorption/^1^H NMR method for the quantitative and at the same time qualitative determination of the products.

### Suitability check of the ^1^H NMR analysis

**Fig. 1:**
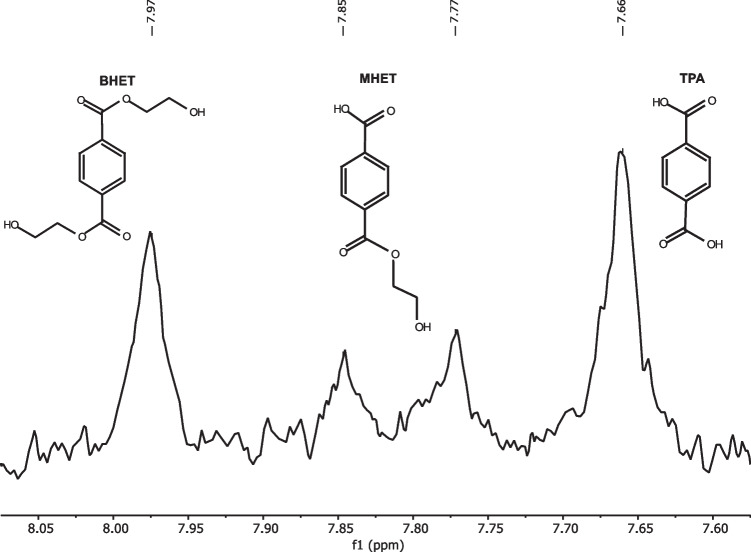
Differentiation of the degradation products to confirm the suitability of ^1^H NMR analysis. A mixture of 500 µM of each product in buffer with pH 9 was analysed on a Spinsolve 60 Carbon Benchtop NMR spectrometer for 10 min. The abscissa shows the chemical shifts of the peaks. A singlet at 7.66 ppm indicates TPA, a doublet at 7.77 and 7.85 ppm indicates MHET, and a singlet at 7.97 ppm belongs to BHET. The degradation products could be clearly distinguished from each other

To verify the suitability of the ^1^H NMR method, a mixture of 500 µM of each of the degradation products was first prepared in a 50 mM glycine–NaOH buffer containing 50 mM NaCl and 10% (v/v) DMSO with pH 9 and measured to check whether it was possible to distinguish the products. In contrast to the other measurements with 8192 scans, this measurement was performed with only 40 scans since the concentration of 500 µM was quite high and thus easier to detect. Consequently, the measurement took only 10 min instead of 16 h. Figure 1 shows the corresponding NMR spectrum with the molecular structures of the products. Two singlets and one doublet can be detected between 8.05 and 7.60 ppm in the aromatic range. The first singlet at 7.66 ppm belongs to the aromatic protons of TPA. The main degradation product MHET shows a doublet at 7.77 and 7.85 ppm because the aromatic is no longer symmetrical. The second singlet at 7.97 ppm indicates BHET. The three products could be clearly distinguished from each other by different aromatic signals, and thus qualitative analysis by ^1^H NMR is confirmed as possible.

### Optimization of the measurement conditions

**Fig. 2:**
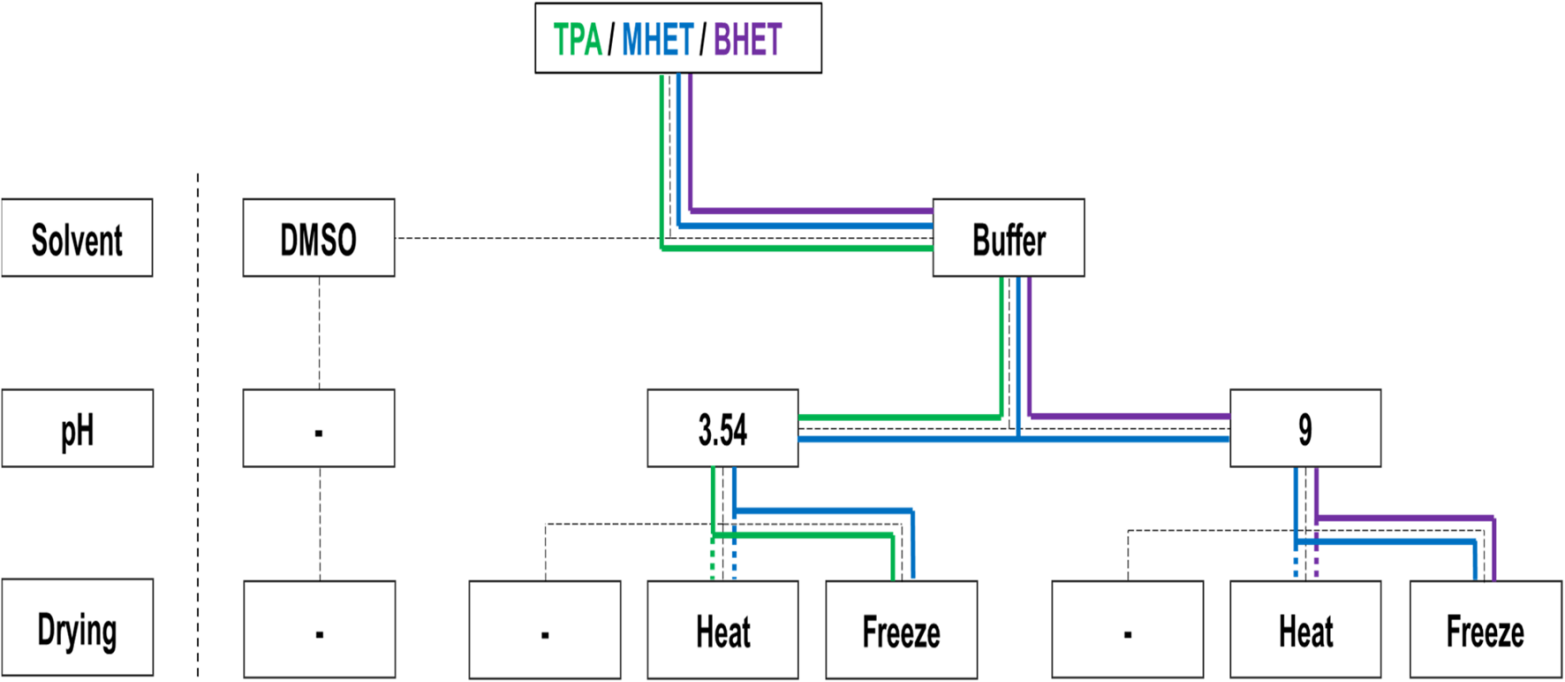
Optimization strategy for absorption and ^1^H NMR measurements. Different solvents, pH values and drying processes were investigated to determine the optimum conditions for the measurement of the degradation products, shown in coloured lines. The best results for TPA and MHET were achieved in buffer pH 3.54 after freeze-drying. For MHET, equal sensitivity was demonstrated in buffer pH 9. The optimal conditions for BHET were at pH 9 in combination with freeze-drying. Nevertheless, sensitivity was also very high in buffer pH 3.54 combined with freeze-drying, so that these conditions proved to be the most suitable

Different pH values, drying methods and solutions were tested to establish the optimal conditions for the ^1^H NMR and absorption measurement and to determine the sensitivity. Since the products are insoluble or sparingly soluble in aqueous solutions and therefore difficult to measure with ^1^H NMR as well as with bulk absorption at lower concentrations, the samples were dried to subsequently dissolve them in a suitable solvent. Both freeze-drying and heat-drying were carried out for this purpose. During drying, TPA crystallized out due to the pH of the buffer being too high at pH 9 and thus could not or only barely be measured. Therefore, the buffer was acidified to the pKs of TPA (pH 3.54) with 1 M HCl before drying. After drying, the products were taken up in the solvent DMSO; the dilution series was prepared and then analysed by ^1^H NMR and bulk absorption measurement. An overview of the optimization of the parameters can be found in Fig. 2. The corresponding experimental data of the optimization tests regarding different solvents, pH values and drying processes can be found in the supplementary material. The clearest spectra of the degradation products and thus the highest sensitivity could be detected directly in DMSO without drying, so even concentrations of 1 µM were not a problem. Moreover, the absorbance values were much higher compared to the measurements of the products in buffer. However, this method will be used in the future for the detection of the enzymatic activity of PETase and MHETase as well as PETase homologs, so the reaction buffer proved to be indispensable. The spectrum of the lowest concentration of MHET could be determined with the freeze-drying procedure, with no differences between the two pH values. BHET showed the best result at pH 9 in combination with freeze-drying. Since TPA crystallized at pH 9 and could not be measured by either method, the optimum conditions for this product could be determined to be pH 3.54 in combination with freeze-drying or heat-drying. In general, both drying methods showed little difference, yet each method has its advantages and disadvantages. While freeze-drying requires more materials such as nitrogen and is therefore more expensive, heat-drying requires considerably more time and could possibly destroy components, which was not the case with PET, however. Also, the pH values did not show much difference for MHET and BHET, but for TPA, only pH 3.54 could be used. Since TPA constitutes an important part of the degradation products, the optimal conditions for the measurement of the mixture were the buffer with pH 3.54 in combination with freeze or heat drying, as the sensitivity was very high for the 3 products in this case. The absorption measurement also provided the best results under these conditions.

### Sensitivity limits for the combined absorption/^1^H NMR method

**Fig. 3:**
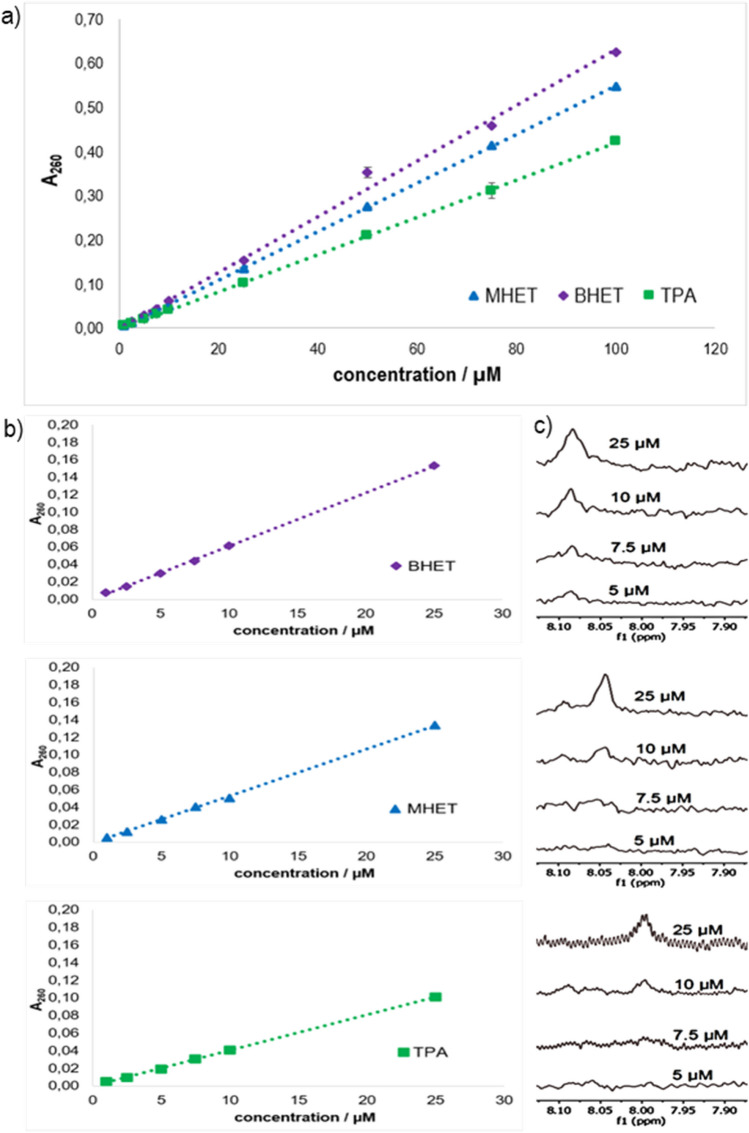
Determination of the sensitivity of the combined absorption/^1^H NMR method. The samples were prepared in buffer with pH 3.54, then freeze-dried and taken up in DMSO. Double determinations were carried out for bulk absorbance. Absorbance profiles of 100 µM (**a**) and 25 µM (**b**) of TPA, MHET and BHET at 260 nm were determined by measurements on a spectrophotometer with error bars indicating standard deviation between 0.004 and 0. The detection limit for the absorption method was between 2.5 and 5 µM for all products. The corresponding spectra (**c**) were recorded on a Spinsolve 60 Carbon Benchtop NMR spectrometer for 16 h. A dilution series was measured for each degradation product to determine the detection limit. For TPA (bottom) and MHET (middle), the detection limit was between 5 and 10 µM, for BHET (top) below 5 µM

Bulk absorbance measurements were carried out in 1-cm-gap UV-transparent cuvettes using a spectrophotometer. For the measurements, the established optimal conditions were applied, so that the samples in buffer with pH 3.54 were freeze-dried and taken up in DMSO. As can be seen in Fig. 3, linear absorption profiles at 260 nm were established for the main products MHET and TPA as well as for BHET. Duplicate determinations were made for each concentration. The coefficients of determination at a concentration of 25 µM obtained by linear regression are *R*^2^ = 0.9992 for MHET, *R*^2^ = 0.9994 for BHET and *R*^2^ = 0.9996 for TPA, so that the measured values show an almost perfect linear relationship, which confirms the suitability of the method. The standard deviation values were between 0 and 0.004. Based on the slope of the calibration line, the different extinction coefficients could be determined to be 5400 M^−1^ cm^−1^ for MHET, 6100 M^−1^ cm^−1^ for BHET, and 4000 M^−1^ cm^−1^ for TPA. The amount of product can be slightly underestimated or overestimated by the absorbance measurement, depending on which extinction coefficient is used and if a product is more present in the mixture. Since MHET represents the major part of degradation products, the extinction coefficient of 5400 M^−1^ cm^−1^ is most appropriate. The detection limit for the absorption method was between 2.5 and 5 µM for all products. The highest absorption values were measured for BHET, closely followed by MHET. TPA had the lowest values.

The ^1^H NMR spectra shown in Fig. 3 represent the detection limits of the degradation products with the settings used on the Magritek Spinsolve 60 Carbon Benchtop NMR spectrometer as described above. These measurements were carried out with the same sample preparation as for bulk absorption, so that degradation products in buffer with pH 9 were freeze-dried and taken up in DMSO. For MHET, the detection limit is between 5 and 10 µM, as well as for TPA. The spectra of BHET show a higher sensitivity so that even at 5 µM, a clear peak can still be detected, and the detection limit is thus below 5 µM. The different solvents can cause the ppm values to shift. For example, the spectra of the products dissolved in the buffer are highly field shifted whereas the peaks of the products dissolved in DMSO after drying are low field-shifted. Nevertheless, the shift remains the same for all 3 components, so that BHET has the highest ppm value, followed by MHET and then TPA, as can be seen in Fig. 3. Furthermore, the peak of MHET represents a singlet instead of a doublet, which can be attributed to a superposition of the peaks or the solvent change.

## Discussion

To be able to use the combined method in the best possible way for future recycling strategies, further optimization is still required, especially with regard to the efficacy and efficiency of the enzymes.

### Limitation of the standard methods

The standard methods for measuring PET biodegradation, such as HPLC, are accurate and sensitive methods, but they are often based on endpoint measurements and thus do not provide information about enzyme behaviour over time (Son et al. 2019). Kinetic studies are essential for understanding enzyme function, as reaction rates can decrease over time for some enzymes. The decrease in reaction rate can be explained, for example, by substrate consumption, inhibition by the resulting products or inappropriate reaction conditions under which the enzymes are unstable (Bisswanger 2017). Visual observations represent simple and rapid methods to obtain an initial overview of PET degradation, but these analyses do not ensure insight into enzyme kinetics either. Various methods with continuous measurements have already been set up, such as fluorescence detection methods, but these are not suitable for larger PET films, such as those found as pollution in the environment (Wei et al. 2012). Also, NaOH titration using a pH–stat as an indicator is not suitable for kinetic analysis, because the intermediate BHET is a neutral product and therefore cannot be detected (Ronkvist et al. 2009). With the absorption method described by Zhong-Johnson et al. (2021) and modified by us, continuous measurement can easily be carried out, because all degradation products contribute to the absorption due to their aromatic rings (Zhong-Johnson et al. 2021). Another positive aspect represents the high sensitivity of 2.5–5 µM for all degradation products, as can be seen in Fig. 3. Furthermore, the bulk absorption method can be adapted to the measurement of degradation of other specific types of plastics. However, this is only possible for those plastics whose degradation products have an aromatic ring that contributes to the absorption. An example is the thermoplastic polystyrene, which consists of the monomer styrene, an unsaturated aromatic hydrocarbon (Danso et al. 2019). Aromatic polycarbonates represent further examples (Wehrmann 2001). Both plastics have aromatics and can thus be quantified by absorbance. Depending on the degradation mechanism of the enzymes involved, the absorbance measurements can be carried out either by increasing the degradation products or by decreasing the aromatic substrate.

According to the literature, the expected amounts of PET degradation products quantified by HPLC after 4 h of reaction with wild-type PETase are about 30 µM for MHET, about 10 µM for TPA and about 2 µM for BHET; hence, the concentration range up to 25–50 µM is of particular interest (Zhong-Johnson et al. 2021). It could be shown that the sensitivity of the absorption measurement with 2.5–5 µM was slightly higher than the ^1^H NMR analysis, which was between 5 and 10 µM for each degradation product. Accordingly, the sensitivity of both methods is sufficiently high to investigate the enzymatic activity of PETase and its homologs in the context of an activity assay.

### Inline measurements using flow cells

If only the presence of PET degradation products is to be determined, the bulk absorbance method is particularly suitable as the measurements can be performed in a few seconds with minimal effort and at low costs, so that rapid measurements during an enzymatic reaction can be used to check whether degradation products are present at all. For example, flow cells can be used for process photometers and allow inline measurement of enzymatically converted products from PET during a controlled automated process in a plant, eliminating the time required for sampling and measurement in the laboratory. However, no continuous measurements with flow cells were carried out as part of this study. Another positive feature is the prevention of contamination of the products. As already shown in other publications, automated continuous monitoring of various parameters using such flow cells is possible. Maierhofer et al. (2021), for example, present a novel inline measurement of ammonia in methanol, for which an ammonia sensor was combined with a flow cell, ensuring efficient measurement under continuous flow (Maierhofer et al. 2021). Jia et al. (2015) present another example of continuous measurement using flow cells for monitoring algea growth by optical density measurements at different wavelength in real time without any sample preparation (Jia et al. 2015). Accordingly, the aromatic degradation products of PET could also be continuously detected by using flow cells for process photometers. After and during the absorption measurement, the product distribution and identification can then be analysed by ^1^H NMR spectroscopy by taking and preparing samples at different times, because continuous measurement is not yet possible. Using the methods established in the current work, the product concentrations in the wastewater after an enzymatic reaction with the PETase could be continuously monitored by bulk absorption measurement using flow cells followed by endpoint ^1^H NMR analysis to identify and distribute the products.

## Conclusion

In this work, we were able to validate benchtop ^1^H NMR spectroscopy as a new method for the qualification of PET degradation products. We suggest the quantitative absorption method coupled with the qualitative benchtop ^1^H NMR method as a modern strategy for monitoring in the automated degradation of PET by enzymes. Enzymatic digestion of PET releases aromatic products with each PETase homolog, so this combined absorption/^1^H NMR method has the great advantage that it can be used universally to determine the activity of all PETase homologs that equally degrade the plastic enzymatically into MHET, BHET and TPA. Furthermore, the bulk absorption method can be adapted to the measurement of degradation of other specific types of plastics that also have aromatic rings. In summary, the methods established in our work can potentially contribute to the development of suitable recycling strategies of PET and other aromatic plastics degraded, for example, by recombinant enzymes immobilized on a suitable membrane in wastewater. As a result, the plastic waste issue in the environment can be counteracted.

### Supplementary Information

Below is the link to the electronic supplementary material.

Supplementary file1 (XLSX 267 KB)

## Data Availability

Data available upon request.
